# Training the Next Generation of Pharmacist Educators (NextGenRxEd): Outcomes from a Longitudinal 4-Year Teaching and Research Program

**DOI:** 10.3390/pharmacy13030058

**Published:** 2025-04-22

**Authors:** Candis M. Morello, Mark Bounthavong, Jan D. Hirsch

**Affiliations:** 1 Skaggs School of Pharmacy and Pharmaceutical Sciences, University of California San Diego, La Jolla, CA 92093, USA; mbounthavong@health.ucsd.edu; 2 School of Pharmacy and Pharmaceutical Sciences, University of California Irvine, Irvine, CA 92697, USA; jdhirsch@uci.edu

**Keywords:** pharmacy education, pharmacy students, pharmacy faculty, training

## Abstract

To combine didactic education with clinical and research experiences that would empower student pharmacists to consider postgraduate training and a career in pharmacy education or academics, in 2009, the Next Generation of Pharmacist Educators (NextGenRxEd) program, a four-year longitudinal education program, was implemented at the University of California San Diego Skaggs School of Pharmacy and Pharmaceutical Sciences. Directed by two pharmacist faculty, a clinician and researcher, student pharmacists were exposed to hands-on experience with clinical management, patient care, and research processes. A post-graduation survey was created and administered in Fall 2024 to evaluate outcomes for all student pharmacists who completed the program. Key performance indicators included the number of pharmacy students proceeding to postgraduate training, research practices, and pharmacist positions involving education and academics. During 2009–2024, 34 student pharmacists completed the NextGenRxEd program; 71% achieved postgraduate education (22 PGY1 residencies and two fellowships). Following quality improvement implementation, this percentage increased from 50% to 89%, for Classes 2009–2015 and 2016–2024, respectively. Overall, the PGY1 match rate was 92%, and 19 abstracts/posters and 10 manuscripts were published, respectively. The post-graduation survey response rate was 85%, with 93% of respondents reporting that they precepted PharmD students; 14% became faculty members, and 66% participated in research. The NextGenRxEd program provides a model whereby student pharmacists are equipped to obtain post-graduate education and pursue a career with a significant role in pharmacy education or academic pharmacy. This program has the potential to be implemented at other pharmacy schools/colleges to benefit faculty and student participants.

## 1. Introduction

Pharmacist educators are a diverse group of pharmacists who play a critical role in the education and training of future pharmacists. They include academic pharmacists (faculty), preceptors, residents, and others with significant roles for student and trainee education. The overarching goal of all pharmacist educators is to prepare future cadres of pharmacists, as stated in the Oath of a Pharmacist, “I will utilize my knowledge, skills, experiences, and values to prepare the next generation of pharmacists” [1]. Aligned with this mission is the American Society of Health-System Pharmacists (ASHP) Section of Pharmacy Educators (SPE) special interest group, which provides pharmacist educators with a catalog of resources supporting those “involved in the training of the pharmacy workforce” [2]. Similarly, academic pharmacists, who generally make up a large proportion of the clinical faculty of pharmacy schools, seek to provide high-quality education and training through pharmacy practice, scholarship, teaching, and mentorship [3,4,5].

As the pharmacist profession evolves to incorporate more patient care responsibilities beyond the traditional compounding and dispensing duties, there is a need for pharmacists to make contributions to the literature through scholarly activities [6,7]. However, preparation of student pharmacists for a career where research and scholarly activities are needed to generate robust empirical evidence of the value of pharmacists have lagged behind this demand.

Developing and encouraging student pharmacists to pursue post-graduate training and a career that includes a significant role as a pharmacist educator or as an academic pharmacist requires a combination of didactics, applications, and experience. The Learning-By-Doing principle provides a framework for meeting these components [8]. A survey administered to United States (U.S.) pharmacy schools reported that the most common methods used to introduce students to pharmacist educator responsibilities and careers in academia were advanced pharmacy practice experiences (APPEs) (96%), structured short-term research experiences (44%), pharmacy organization experiences (44%), and independent studies (42%) [9]. The authors noted that few schools offered longitudinal programs or tracks related to academic pharmacy, although tracks have advantages and are used for training students in other specialized pharmacy practice areas. Moreover, limited time, faculty inexperience, lack of leadership support, and disinterest by student pharmacists were identified as barriers to delivering programs exposing students to careers in academic pharmacy.

Comprehensive training to learn about multiple elements of an academic pharmacist role (e.g., teaching, research, administrative, and patient clinical care) requires training standards on research skills, time dedicated to scholarly activities, and access to mentors with experience in conducting investigations [6]. In addition to pharmacy residency programs, other post-graduate training offerings are available, such as residency certificates addressing single elements (e.g., teaching or research) or more comprehensive fellowship programs covering multiple elements over time [10,11,12,13,14,15]. To date, there are limited examples of long-term longitudinal programs where student pharmacists, prior to graduation, can acquire the necessary skills and experience to pursue a career as a pharmacist educator or an academic pharmacist. Pharmacy school education has focused on the evolving practice of pharmacy from compounding and dispensing to consulting and direct patient care [16]. However, there is a gap in terms of developing a comprehensive educational curriculum and experience to prepare student pharmacists in roles involving academics and research. To address this gap, two faculty members at the University of California (UC) San Diego Skaggs School of Pharmacy and Pharmaceutical Sciences (SSPPS) developed and implemented a pilot, four-year longitudinal student training program to encourage student pharmacists to consider careers as active pharmacist educators or as academic pharmacists—the Next Generation of Pharmacist Educators (NextGenRxEd) program.

Capitalizing on the strengths of an existing pharmacist-run diabetes clinic [17,18]. NextGenRxEd program incorporated the Learning-By-Doing principle, which focuses on learning from one’s own experiences rather than through conventional didactic instructions [8]. Further, pharmacy students would translate lessons from their course work into practice via active clinic management and complete a student-initiated research project. The goal of the NextGenRxEd program was to combine didactic education with robust clinical and research experiences that would empower student pharmacists to consider post-graduate training and a career with a significant role in pharmacy education or in academic pharmacy. As with any new educational program or model, it is important to assess outcomes for students, though this step may be overlooked. Current models of pharmacist education introduce academia in experiential rotations/electives, longitudinal research tracks, or independent studies, but do not evaluate whether these lead to academic careers [9,15]. In this brief report, we provide a description of the NextGenRxEd program, including key improvements made in two phases, and a summary of program outcomes including completion and publication rates, and our student pharmacists’ post-graduate experiences.

## 2. Materials and Methods

### 2.1. NextGenRxEd Program Description

The NextGenRxEd program began following the implementation of the pharmacist-run Diabetes Intense Medical Management (DIMM) “Tune-Up” Clinic at the VA San Diego Health System (VASDHS) in 2009. The DIMM “Tune-Up” Clinic was a collaborative practice that was operated by a clinical pharmacist practitioner and overseen by an endocrinologist [17]. The clinic’s goal was to help patients achieve metabolic control using a holistic approach that provided patient-centered comprehensive medication management, individualized healthy lifestyle practices, and addressed medication adherence barriers [18]. The decision to incorporate the NextGenRxEd program alongside the DIMM “Tune-Up” Clinic was based on the Learning-By-Doing principle [8]. Interested student pharmacists were provided an environment to experience how to manage a pharmacist-run clinic, communicate and provide care to patients, create and implement a related research plan, and apply research findings to inform clinical practice in order to inspire meaningful interest in post-graduate education and eventually a career involving pharmacy education or academia. The program was overseen by two academic pharmacist faculty directors [a clinical pharmacist practitioner director and a health services research director] and included 1-3 student pharmacists from each year of the program (P1 to P4 years), annually.

### 2.2. Phase I—Implementation (2009–2012)

The initial focus of the NextGenRxEd Program in Phase I centered on implementation. A process improvement evaluation was performed to assess the initial implementation period (2009–2012). Throughout the program, students were asked about their experiences and satisfaction with the program and to provide suggestions for optimization. This feedback was collected annually for qualitative analysis. Two key lessons were learned from this initial phase. Initially, faculty selected students to participate in the NextGenRxEd Program if students expressed interest in learning about clinical practice, teaching, and research. However, after a few years, it became apparent that student interest alone did not always yield long-term commitment to the program; hence, a more systematic student identification and learning approach was needed. Students also expressed that their experience was optimized when two student pharmacists from each year were enrolled, and that monthly team meetings helped each cohort learn from the other.

### 2.3. Phase II (2012–Present)

In 2012, based on student and faculty feedback of the program, significant program modifications were employed which started with the entering Class of 2016. Modifications resulted in a more structured and progressive program where student pharmacists improved their skills and acquired more responsibilities as they progressed through the four-year program (Table 1). Each year, two first-year pharmacy (P1) students were interviewed by P2 team members and selected to start the program, and two fourth-year pharmacy (P4) students graduated, resulting in an ongoing cohort of eight student pharmacists per year, with varying roles and responsibilities as outlined in Table 1. We employed additional changes based on student feedback to improve the program cohesiveness. First, we initiated monthly team meetings to ensure that learning objectives were met, and encouraged open discussion for additional learning and collaborative opportunities. We incorporated teaching and modeling soft skills such as communication, professionalism, organization, leadership, and collaboration for our trainees, with each cohort learning from the other. Over four years, student pharmacists were exposed to learning objectives that grew in complexity and responsibility with each successive year. The two faculty directors performed ongoing performance-based evaluation of trainees to ensure learning objective achievement for each P1–P4 year.

### 2.4. Program Evaluation

Program evaluation measures included program completion rate; percent of students completing post-graduate education and certifications; clinical, teaching, and research experiences since graduation; opinion on how experience prepared them for activities related to an active pharmacist educator role in practice or academia; and number of abstracts and publications resulting from research projects. Data for program completion rate and post-graduate education immediately after graduation were based on the institution’s data. The remaining data were collected via a 16-item electronic survey tool that was sent to all graduates of all NextGenRxEd cohorts (classes 2010 through 2024) in the fall of 2024 via text and email messages, with two follow-up reminders spaced at two-week intervals. The survey included questions regarding respondent demographics; post-graduate education, certifications, and practice settings; clinical, teaching, and research experiences since graduation; and opinion on how the NextGenRxEd experience had prepared them for activities related to an active pharmacist educator role in practice or academia. A final open-ended question queried the main value of participation in the program over their career so far. Where possible, questions were modeled on the AACP Graduating Student Survey (e.g., demographics, practice sites), while questions regarding post-graduate experiences and opinion of the program preparation value were based on key elements of the program [19]. Opinions were assessed on a four-point Likert scale (strongly agree to strongly disagree), and thematic content analysis was used to summarize open-ended question responses. QuestionPro was utilized to field the survey and for data analysis to summarize responses. The survey was determined to be exempt from Institutional Review Board (IRB) requirements by both UC Irvine and UC San Diego IRB offices.

## 3. Results

### 3.1. Overall Findings

Between 2009 and 2024, a total of 35 student pharmacists (89% identified as female) were selected to be part of the NextGenRxEd Program. Program completion rate was 97% (n = 34), as one student from the Phase 1 cohort withdrew for personal reasons. Overall, 71% (24/34) of student pharmacists completed post-graduate education after completion of the NextGenRxEd Program (22 PGY1 residencies and 2 fellowships). This percentage increased from 50% (8/16) in the Phase 1 cohort to 89% (16/18) in the Phase 2 cohort.

### 3.2. Survey Responses

**Figure 1:**
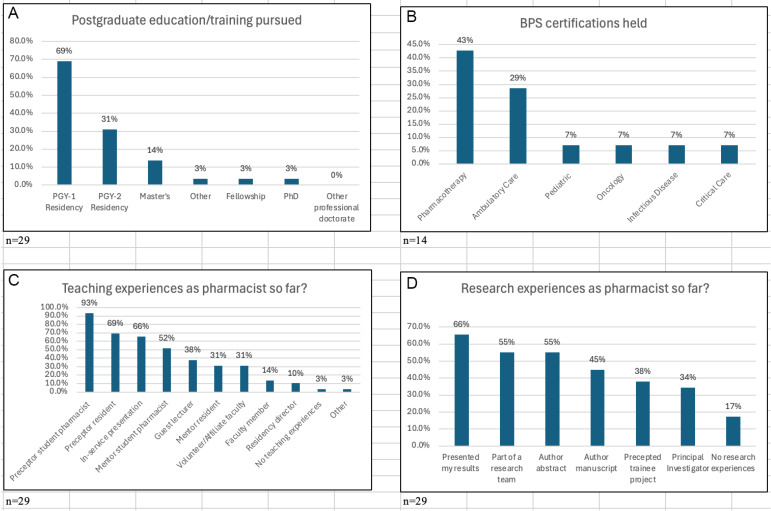
Summary of the outcomes of NextGenRxEd graduates’ variety of experience related to post-graduate training (**A**), Board of Pharmacy Specialties certifications (**B**), types of teaching (**C**) and research (**D**). PGY-1, Post-Graduate Year 1; PGY-2, Post-Graduate Year 2; BPS, Board of Pharmacy Specialties.

The survey response rate was 85%, with the majority (86%) of respondents identified as female and between 26 and 40 years of age. At the time of the survey, over half (55%) worked in a hospital setting, followed by others such as clinics (48%), community (38%), academia (21%), and managed care (14%). Approximately one-third had completed, or were currently pursuing, a PGY2 residency (Figure 1A), and nearly half (48%) held or had held a certification from the Board of Pharmacy Specialties (BPS), with Pharmacotherapy (43%) and Ambulatory Care (29%) as the most reported (Figure 1B). Other types of certifications reported were Certified Diabetes Care and Education Specialist (n = 2), Advanced Practice Pharmacist (n = 1), Diplomate, Pharmacy Leadership Academy (n = 1), and American Hypertension Specialist Certification Program—Certified Hypertension Clinician (n = 1).

Virtually all (97%) of the respondents reported having teaching experiences, and the majority (83%) reported having research experiences during their career as a pharmacist. The proportion of respondents having had various types of teaching experiences ranged from precepting and mentoring pharmacy students and other trainees (range: 31–93%), to providing in-service presentations or similar (66%), to more formal academic roles as faculty members, residency directors, or guest lecturers (range 10–38%) (Figure 1C). Research experiences included presenting results of their own research or quality improvement project (66%) and precepting resident or student research projects (38%), and approximately one-half had served as part of a research team or had been an author on a publication or professional meeting abstract (Figure 1D).

The vast majority of respondents agreed or strongly agreed that the NextGenRxEd experience better prepared them for activities related to the roles of pharmacist educator or academic (Table 2). Greater than three-quarters of respondents agreed or strongly agreed that the program prepared them to perform clinical and research activities, as well as obtaining post-graduate training and qualifying for their first pharmacy practice position. Identical ratings were reported regarding how the program had positively impacted respondent confidence in the same activities and responsibilities in Table 2. Themes that emerged in the open-ended question regarding the main value of their participation in the NextGenRxEd program were related to the following: research (n = 14), clinical patient care (n = 13), clinic management (n = 8), the value of pharmacist in clinic setting (n = 5), faculty mentorship (n = 3), and opening employment doors (n = 2). Thus far, the students who participated in the NextGenRxEd Program have been involved with presenting 19 abstracts/posters, with 24 student pharmacists, and publishing 10 manuscripts, with 25 program graduates, based on the students’ research projects [17,18,20,21,22,23,24,25,26,27].

## 4. Discussion

The NextGenRxEd program’s purpose was to allow student pharmacists to better understand, and thus consider, post-graduate training and a career with a significant role in pharmacy education or academic pharmacy. The success of the program has been demonstrated via the high completion rate of the 4-year program; approximately three-quarters of students have completed post-graduate training; nearly all have had teaching experience since graduation, some with formal academic roles as a faculty member, residency director, or guest lecturer; and most have had some research experience after graduation, including precepting residents and students, through to results dissemination via abstracts or manuscripts.

Notably, from 2009–2024, 24 of our NextGenRxEd program participants applied to the ASHP Match for a PGY1 residency, and 22 (92%) matched. This match rate is higher than the ASHP national reported match rate of 71% and our UC San Diego SSPPS match rate of 79% over the same time period (2009–2024) [28]. In comparison, Slazak and colleagues developed The Scholars Program for P3 and P4 student pharmacists interested in pursuing post-graduate education, and compared ASHP match rate outcomes [29]. The Scholars Program consisted of an elective coursework encompassing areas of clinical practice, teaching, leadership, research, and scholarship. Similarly to our program, their match rate from 2013–2019 was 91% (58/64 pharmacy students). Despite having a high pharmacy residency match rate, The Scholars Program did not evaluate outcomes beyond PGY1 match rates nor post-graduation teaching and research experiences. There is great interest in understanding the career trajectory after post-graduate training. In our evaluation of the program, we reported that pharmacy students who completed a 4-year longitudinal training program (NextGenRxEd) went on to become pharmacist educators and academics. Using the Learn-by-Doing principle [8], our pharmacy students were able to cultivate their initial interests through practice and discovery, culminating in a robust student-initiated research project.

In our follow-up survey that was administered in November 2024, a large proportion of pharmacy school graduates (1 to 15 years post-graduation) mostly agreed that their NextGenRxEd experience prepared them for activities related to a pharmacist educator or academic. Specific activities such as the dissemination of their research projects at professional meetings and publication of peer-reviewed manuscripts were critical factors to the pharmacy students’ professional development.

An important positive externality of the NextGenRxEd program was its benefits to participating faculty. Faculty act as academic role models to student pharmacists by teaching, conducting research, performing scholarly activity, publishing/disseminating their work, and providing patient care, while empowering their students to obtain and use these skills. The faculty director experiences are directly related to the fulfillment of their own academic pursuits of teaching, research, scholarly activity, and clinical care. This alignment of incentives may be one that faculty struggle with in their teaching programs, and in this NextGenRxEd model, it becomes a natural and key point for sustaining the program overtime.

Implementation of the NextGenRxEd program at other academic institutions would potentially increase the number of pharmacists who would be prepared to make substantial contribution to the scholarly activities of pharmacists. This would help to meet the demand of the evolving pharmacist profession through the generation of robust empirical studies that support the value of the pharmacist in the future complex healthcare setting. The NextGenRxEd program could be exportable to other pharmacy schools in the U.S. given that the necessary resources to implement this program are likely available. Most schools/colleges of pharmacy have the necessary faculty needed to create and run the program: clinical pharmacist specialists and health services researchers who can either be a Pharm.D., Ph.D., masters level research degree, or a combination. There is alignment with faculty needs since many are required to fulfill some level of teaching, scholarly activity, and clinical practice. For both clinic management objectives and the research project (progression from design, data collection, data management, analysis, and reporting), the NextGenRxEd program can be transferable and implemented in any type of clinical practice or specialty area, including ambulatory, inpatient, community, and managed care, and telemedicine, or wherever the faculty member maintains a practice. The research elements will need to incorporate fundamentals such as constructing a testable research question, generating a hypothesis, data collection and analysis, synthesis and interpretation, and dissemination and presentation, all of which are vital components to a pharmacy faculty’s scholarship [30]. Adaption of the NextGenRxEd program may be needed at pharmacy schools where some or all the elements are unavailable. In these cases, we encourage that the program focuses on providing pharmacy students with the protected time and experience to conduct their research study. This is a vital element that will instill upon the pharmacy student much-needed experience, which they can apply to their future roles as pharmacist educators.

Moreover, the NextGenRxEd program meets Accreditation Council for Pharmacy Education (ACPE) education requirements of co-curricular, IPPE, and APPE hours, and is valuable for those students seeking post-graduate experience or pharmacist positions with a significant academic role. The number of students can also be tailored to the faculty percent effort. Currently, in our program, faculty effort is approximately 5% due to the Clinical Director’s significant administrative duties, which is an important reason for our low number of students (2 pharmacy students per cohort for a total of 8 pharmacy students per year). Expanding the NextGenRxEd Program to other pharmacy faculty and clinics is one of our long-term goals. Modified learning objectives best suited to the clinical practice and site can easily be included alongside the clinic management, research, and direct patient care skills instruction and experiences.

There were several limitations to our analysis of the NextGenRxEd Program. First, implementation of the NextGenRxEd Program was performed at the VASDHS, which is an innovative medical facility where pharmacists can operate as providers under a scope of practice [31]. Not all pharmacy students at other institutions will have this opportunity to observe clinical pharmacists operating at their highest level. Moreover, our facility also provided us with ample support in research investigations, which may not be available to pharmacy students at other institutions. Regardless, we believe that most pharmacy schools will have ample faculty willing to elevate their skills and knowledge to train the next generation of pharmacist educators and academics using the principles of the NextGenRxEd Program. Next, we recognize this is a single-site evaluation of a small number of students, and thus, results are not generalizable to other institutions and instead are intended to help others consider similar programs. Additionally, there is selection bias in student selection, since only students interested in the program could be selected, as is the case in many student experiences. Our survey was administered in November 2024, which can introduce recall bias among those who completed the NextGenRxEd Program as early as 2009 [32]. Although our response rate was high at 85%, alumni of the program may have varying levels of reflection and bias the further they are removed from their graduation. Moreover, older alumni may also have more opportunities to apply their skills to activities related to pharmacist educators and academics. A final limitation was the survey design, in that it was a non-validated survey and was sent by faculty of the NextGenRxEd Program; thus, graduate responses may have been artificially positive. Future studies should be designed to evaluate long-term outcomes at systematic timepoints post-graduation for each participant and be fielded to promote anonymous responses. While the description of these program outcomes may help other institutions create a similar program, a more detailed toolkit by Haines and colleagues, which was derived from contributions of U.S. colleges/schools of pharmacies to prepare student pharmacists for careers in academia and education, would be a useful companion for designing specific curricular elements [33].

## 5. Conclusions

The NextGenRxEd program is an innovative, pilot, pharmacy education model, comprising elements (education, scholarly activity, research, teaching, and dissemination) that generate mutual benefits for both the faculty and trainees involved. Resources needed are considerable; however, these payoffs could be very important to the pharmacy field. Early investments, such as this program, are critical and necessary if we want to ensure a robust and healthy supply of actively involved pharmacist educators in the future. The NextGenRxEd program provides a detailed framework for other institutions to adopt.

## Figures and Tables

**Table 1:** Longitudinal program components: Roles and duties of student cohorts, learning objectives, and key performance indicators, 2012 to current.

Trainee Year & Role	Duties	Learning Objectives	Key Performance Indicator	IPPE/CO-CURRICULAR	APPE	Research
P1 Clinic Manager	• Train on clinic management skills • Oversee clinic administrative operations • Communicate with patients prior to appointments • Shadow clinical practitioner in DIMM Clinic • Shadow P2 cohort	• Describe the process for obtaining a WOC appointment at the VASDHS • Be able to explain, discuss and apply all of the VASDHS training requirements: initial and annual • Employ OSCE-related skills including patient communication and education • Collect, analyze and interpret laboratory values • Demonstrate key clinic management functions	• WOC appointment approval • Training Completion certificate. • IPPE assessment and reflection of the experience • Clinic preparation operations 2 h per week	Yes, 24 h available.	No	No
P2 Mentor for P1	• Recruit, interview, and train next P1 cohort • Develop a research plan for their project • Submit research plan to IRB	• Conduct recruitment, interviews, and selection of two new P1 team members • Train P1 clinic managers • Create an IRB approvable research proposal for human subjects	• Recruitment and training process reviewed and evaluated by Clinical Director • P2 students take over clinic management • IRB approval	No	No	Yes
P3 Mentor for P2	• Train on VASDHS research rules and regulations • Perform research project • Provide research updates at monthly team meetings	• Describe the process for obtaining a research WOC appointment at the VA • Collect research project data • Analyze research project data • Describe research methods, results and interpret in a presentation to the team	• Research WOC appointment approval. • Research presentation evaluated and critiqued by Directors and student members.	No	No	Yes
P4 Mentor for P3	• Complete research project • Present research findings at a monthly team meeting • Submit abstract to a national meeting • Draft manuscript for graduation credit, and submit for publication as P4 or as alumnus, depending on timing	• Participate in APPE clinical opportunity (as available) • Conceptualize an abstract and poster based on research findings • Submit abstract to professional meeting • Present poster at a school-wide poster session • Present poster at national/international meeting • Write an initial manuscript draft	• APPE assessment and reflection of the experience • Abstract and poster reviewed and approved by Clinical and Research Directors • Poster reviewed and critiqued by school faculty • Abstract evaluated by professional meeting reviewers • Manuscript evaluated by Clinical and Research Directors	No	Yes	Yes
Alumni	• N/A	• Construct a manuscript for peer review submission	• Accepted for review in a peer-reviewed journal	No	No	No

Definitions: DIMM, Diabetes Intense Medical Management; VASDHS, Veteran Affairs San Diego Healthcare System; WOC, Without Compensation; OSCE, Objective Structured Clinical Examination; IRB, Institutional Review Board; I/APPE, Introductory/Advanced Pharmacy Practice Experience.

**Table 2:** Respondent agreement ratings regarding degree to which the DIMM clinic experience better prepared them (n = 29).

Statement	Strongly Agree	Agree	Disagree	Strongly Disagree	Unable to Comment
Pursuing postgraduate education *	59%	35%	0%	0%	7%
Educating learners (e.g., students, residents) *	45%	52%	4%	0%	0%
Educating other health care professionals (e.g., nurses, physicians) *	38%	55%	4%	0%	4%
Conducting research or quality improvement projects	72%	14%	0%	0%	14%
Clinic management *	45%	28%	4%	0%	24%
Patient communication and interaction	45%	41%	4%	0%	10%
Applying clinical guidelines for patient care *	45%	45%	4%	0%	7%
Obtaining postgraduate training/education	69%	14%	0%	0%	17%
Qualifying for my FIRST pharmacy practice position *	38%	38%	4%	0%	21%

* Does not sum to 100% due to rounding error.

## Data Availability

Data are found in the corresponding tables.

## References

[1] American Association of Colleges of Pharmacy (2021). Oath of a Pharmacist|AACP. https://www.aacp.org/resource/oath-pharmacist.

[2] American Society of Health-System Pharmacists (2025). ASHP Section of Pharmacy Educators. https://www.ashp.org/pharmacy-educators.

[3] International Pharmaceutical Federation Academic Pharmacy. https://www.fip.org//academic-pharmacy.

[4] Brooks A.D. (2009). Considering academic pharmacy as a career: Opportunities and resources for students, residents, and fellows. Curr. Pharm. Teach. Learn..

[5] Nutescu E.A., Engle J.P., Bathija S., Grim S.A., Chan J., Mucksavage J.J., Ohler K.H., Tesoro E.P., Thielke J.J., Shapiro N.L. (2014). Balance of academic responsibilities of clinical track pharmacy faculty in the United States: A survey of select American College of Clinical Pharmacy Practice and Research Network Members. Pharmacotherapy.

[6] Deal E.N., Stranges P.M., Maxwell W.D., Bacci J., Ashjian E.J., DeRemer D.L., Kane-Gill S.L., Norgard N.B., Dombrowski L., Parker R.B. (2016). The Importance of Research and Scholarly Activity in Pharmacy Training. Pharmacotherapy.

[7] National Health Service Report of a UK Survey of Pharmacy Professionals’ Involvement in Research. https://www.england.nhs.uk/long-read/report-of-a-uk-survey-of-pharmacy-professionals-involvement-in-research/.

[8] Reese H.W. (2011). The Learning-By-Doing Principle. Behav. Dev. Bull..

[9] Haines S.L., Dy-Boarman E.A., Clifford K.M., Summa M.A., Willson M.N., Boyle J.A., Peeters M.J. (2017). Methods Used by Colleges and Schools of Pharmacy to Prepare Student Pharmacists for Careers in Academia. Am. J. Pharm. Educ..

[10] Darko W., Seabury R.W., Miller C.D., Spinler S.A., Probst L.A., Cleary L.M., Kelly C., Kufel W.D. (2021). Implementation of a formal pharmacy residency research certificate program. Am. J. Health Syst. Pharm..

[11] Weeda E.R., Weant K.A. (2021). Development of a Pharmacy Residency Research Certificate Program. Hosp. Pharm..

[12] Wahl K.R., Margolis A., Lintner K., Hartkopf K., Martin B. (2014). Impact and application of material learned in a pharmacy residency teaching certificate program. Am. J. Pharm. Educ..

[13] Brown J.N., Tiemann K.A., Ostroff J.L. (2014). Description of a medical writing rotation for a postgraduate pharmacy residency program. J. Pharm. Pract..

[14] Wanat M.A., Fleming M.L., Fernandez J.M., Garey K.W. (2014). Education, Training, and Academic Experience of Newly Hired, First-Time Pharmacy Faculty Members. Am. J. Pharm. Educ..

[15] Harrington E.A., Gawronski K.M. (2018). An advanced pharmacy practice experience in academia: More benefit than burden!. Curr. Pharm. Teach. Learn..

[16] Bloom T.J., Kebodeaux C., Munger M., Smith M.D., Stutz M., Wagner J. (2025). A Narrative Review of Pharmacy Identity and the PharmD Experiment. Am. J. Pharm. Educ..

[17] Morello C.M., Christopher M.L.D., Ortega L., Khoan J., Rotunno T., Edelman S.V., Henry R.R., Hirsch J.D. (2016). Clinical Outcomes Associated with a Collaborative Pharmacist-Endocrinologist Diabetes Intense Medical Management “Tune Up” Clinic in Complex Patients. Ann. Pharmacother..

[18] Hirsch J.D., Kong N., Nguyen K.T., Cadiz C.L., Zhou C., Bajorek S.A., Bounthavong M., Morello C.M. (2021). Improved Patient-Reported Medication Adherence, Patient Satisfaction, and Glycemic Control in a Collaborative Care Pharmacist-Led Diabetes “Tune-Up” Clinic. Int. J. Environ. Res. Public Health.

[19] American Association of Colleges of Pharmacy Alumni Survey (2022). 2022 National Summary Report.

[20] Hirsch J.D., Bounthavong M., Arjmand A., Ha D.R., Cadiz C.L., Zimmerman A., Ourth H., Morreale A.P., Edelman S.V., Morello C.M. (2017). Estimated Cost-Effectiveness, Cost Benefit, and Risk Reduction Associated with an Endocrinologist-Pharmacist Diabetes Intense Medical Management “Tune-Up” Clinic. J. Manag. Care Spec. Pharm..

[21] Morello C.M., Nguyen T., Tao L., Hirsch J.D. (2020). Improved Glycemic Control Outcomes Regardless of Mental Health Disorders in a Pharmacist-Endocrinologist Diabetes Intense Medical Management (DIMM) “Tune Up” Clinic. Ann. Pharmacother..

[22] Morello C.M., Lai L., Chen C., Leung C.M., Hirsch J.D., Bounthavong M. (2022). Longitudinal Effects on Metabolic Biomarkers in Veterans 12 Months Following Discharge from Pharmacist-Provided Diabetes Care: A Retrospective Cohort Study. Pharmacy.

[23] Luli A.J., Awdishu L., Hirsch J.D., Watanabe J.H., Bounthavong M., Morello C.M. (2021). Transferring Key Success Factors from Ambulatory Care into the Community Pharmacy in the United States. Pharmacy.

[24] Bounthavong M., Medina A., Wallace B.M., Sepassi A., Morello C.M. (2024). Impact of increasing number of mental health conditions on healthcare costs and resource utilization among individuals with type 2 diabetes: A cross-sectional study. J. Pharm. Health Serv. Research.

[25] Yip O., Du E., Morello C.M., Bounthavong M. (2024). Comparison between in-person, telehealth, and combination visits among veterans treated in a pharmacist-led diabetes management clinic. J. Am. Pharm. Assoc..

[26] Morello C.M., Rotunno T., Khoan J., Hirsch J.D. (2018). Improved Glycemic Control with Minimal Change in Medication Regimen Complexity in a Pharmacist-Endocrinologist Diabetes Intense Medical Management (DIMM) “Tune Up” Clinic. Ann. Pharmacother..

[27] Morello C.M., Awdishu L., Lam S., Heman A., Bounthavong M. (2024). Sodium-Glucose Cotransporter-2 Inhibitors versus Glucagon-Like Peptide 1 Receptor Agonists Effects on Kidney and Clinical Outcomes in Veterans with Type 2 Diabetes. Kidney360.

[28] American Society of Health-System Pharmacists Statistics of the Match. 6 January 2025. https://natmatch.com/ashprmp/stats.html.

[29] Slazak E.M., Prescott G.M., Doloresco F., Woodruff A.E., Prescott W.A. (2020). Assessment of a Scholars Program Designed to Enhance Pharmacy Students’ Competitiveness for Postgraduate Residency Training. Am. J. Pharm. Educ..

[30] Pickard A.S. (2006). Towards supporting scholarship in research by clinical pharmacy faculty. Pharm. Pract. (Granada).

[31] Ourth H., Groppi J., Morreale A.P., Quicci-Roberts K. (2016). Clinical pharmacist prescribing activities in the Veterans Health Administration. Am. J. Health. Syst. Pharm..

[32] Althubaiti A. (2016). Information bias in health research: Definition, pitfalls, and adjustment methods. J. Multidiscip. Healthc..

[33] Haines S.L., Summa M.A., Peeters M.J., Dy-Boarman E.A., Boyle J.A., Clifford K.M., Willson M.N. (2017). Toolkit for US colleges/schools of pharmacy to prepare learners for careers in academia. Curr. Pharm. Teach. Learn..

